# Health Tourism—Subject of Scientific Research: A Literature Review and Cluster Analysis

**DOI:** 10.3390/ijerph20010480

**Published:** 2022-12-28

**Authors:** Michał Roman, Monika Roman, Monika Wojcieszak-Zbierska

**Affiliations:** 1 Department of Tourism, Social Communication and Consulting, Institute of Economics and Finance, Warsaw University of Life Sciences, Nowoursynowska 166, 02-787 Warsaw, Poland; 2 Department of Logistics, Institute of Economics and Finance, Warsaw University of Life Sciences, Nowoursynowska 166, 02-787 Warsaw, Poland; 3 Faculty of Economics, Poznań University of Life Sciences, Wojska Polskiego 28, 60-637 Poznań, Poland

**Keywords:** literature review, trend, cluster analysis, co-word, tourism, tourism economics

## Abstract

The purpose of this article is to identify main research areas in health tourism in scientific research. The data used in this analysis span from 2000 to 2022, was retrieved from the Web of Science database, and comprises a total of 1493 bibliometric records of publications. The paper includes both a quantitative and a qualitative analysis. The following four main research areas were identified based on the results: (1) patient satisfaction built upon trust; (2) health impacts of the destination (including the economic aspect, which plays a decisive role in choosing a tourism destination); (3) health behavior as a major part of human activity; and (4) traveling with a view to regain one’s health. Note that the limitations of this study—which mostly affect the methodological part—need to be taken into consideration. This is the consequence of the selected publication database and of the search criteria used, such as the publication year or language.

## 1. Introduction

Travel and tourism, which keeps evolving, is the world’s largest service industry [1,2]. It drives an increase in Gross Domestic Product (GDP) in destination countries, while also strongly contributing to their government’s tax income [3]. The travel and tourism sector is worth USD 7.6 trillion and accounts for more than 10% of the global GDP, 7% of total international trade, and 30% of service exports around the world. Income from tourism also provides an important currency exchange engine for countries worldwide, thus becoming an enabler of economic growth and investments in a number of other sectors. In 2016, tourism grew by 3.1%, which is 2.5% above the growth rate of the global economy [4].

As the third largest socioeconomic activity in the European Union, tourism is a major contributor to its gross domestic product and employment. While Europe is the world’s most popular tourism destination, it is not the fastest developing region at a global level. It has experienced a decline in its market share, measured by the visits of and income from international tourists [5]. In 2018, Europe had the world’s largest number of international visitors (713 million, i.e., more than half of the global total) and saw a growth rate of 6% [2].

Tourism is a sector whose income keeps increasing [6]. It forms a major part of many economies, while also having a considerable impact on human (including inter-generational) relationships and on global international connections. At a global level, tourism is viewed as a sector of extreme importance, as it brings crucial benefits to the economy [7]. Each year, it generates ca. 10% of the global GDP, making it the third largest sector of the economy [8]. In this context, it mostly performs a macroeconomic function, as it stimulates the socioeconomic development of a country by adding value, which translates into an increase in gross domestic product [9,10].

Tourism is among the largest and fastest developing sectors of the global economy [11]. By creating jobs, driving revenues from exports, and boosting investments and infrastructural development, tourism both directly and indirectly contributes in a significant way to socioeconomic processes. Note, however, that the COVID-19 pandemic had a disastrous effect on tourism development worldwide [12].

Even prior to COVID-19, global tourism was facing a number of crises. The main breakthrough events from 2000–2015 include the 9/11 terrorist attacks, the outbreak of the Severe Acute Respiratory Syndrome (SARS) in 2003, the global economic crisis in 2008/2009, and the Middle East Respiratory Syndrome (MERS) in 2015 [13].

However, none of the crises listed above resulted in a prolonged global decline in tourism development. Only SARS (−0.4%) and the global economic crisis (−4.0%) entailed a drop in the number of international airline travelers. This would suggest that tourism, as a system, is robust to external shocks. However, there is overwhelming evidence that the consequences of and recovery from the COVID-19 pandemic will be of an unprecedented nature. This is why health tourism has recently become increasingly popular. Society needs some rest and support in the physical, mental, emotional, and spiritual dimensions, which members can find in a number of locations, including rural areas [13].

Tourism has a series of important social functions [14]. Because of the number of purposes it serves, there are ten to twenty classifications of tourism traffic, which are additionally sub-divided into subtypes, kinds, or specific forms [15].

This includes health tourism, often referred to as therapeutic tourism. Różycki [16] identifies two more alternative terms: spa tourism and curative tourism (sometimes, in a broader sense, also referred to as health tourism). Although known for quite a long time, it has become particularly popular over the recent years, and is intended to recover and revitalize the body [17].

Indeed, as people are always in a hurry, they look for ways to alleviate the stress and take care of their mental and physical health [18]. The increased interest in healthy lifestyles, combined with physical and intellectual activity, has directly contributed to changing the existing patterns of spending free time [19]. Therefore, the purpose of this article is to identify main research areas in health tourism in scientific research based on the current literature review.

## 2. Literature Review on Health Tourism

Society demonstrates a growing health awareness [20,21]. Health is believed to be the most important and most precious thing for human life and development, and it can be neither purchased from nor sold to another person [22]. It represents individual wealth and a private value attributed to a particular human being [23]. In order for working people to maintain their health in physical, mental, spiritual (related to individual and social awareness), and social terms, they need to relax in their free time, because, otherwise, their bodies may become weaker and exposed to illnesses in the long run [24].

Claiming that “health is something you either have or do not have” is overly simplistic [25]. Health is something that needs to be taken care of on a continuous basis; people must seek their well-being, have their illnesses (if any) cured, make their bodies fitter, and—first of all—prevent diseases [26]. The increased interest in healthy lifestyles, combined with physical and intellectual activity, contributes directly to changing the existing patterns of spending free time. This means shifting from the 3S (Sun, Sea, Sand) model to the 3E (Entertainment, Excitement, Education) and 3A (Action, Amusement, Adventure) models [27].

As society ages, tourists look for diverse forms of active leisure that will improve their physical and mental condition while allowing their bodies to recover [28]. As today’s society becomes increasingly wealthy, people demonstrate greater demand for luxury goods and services related to improving their health condition. The increased interest in health tourism is viewed as one of the key developments witnessed in the market for tourism services [29]. Table 1 presents some selected definitions of health tourism.

Health tourism is a combination of active leisure, prevention, and treatment [38]. Could it become an important driver of rural development? Indeed, it should develop on environmentally sound areas and has a direct impact on the economic development of the territory concerned [39].

The forms of health tourism are shaped and directly implied by the motives behind it, including [40]:

- post-illness and post-trauma recovery,
- the desire to remove the adverse consequences of stress,
- anti-ageing and beauty treatments (including plastic surgery),
- fighting addictions,
- the decision to improve one’s health condition by undergoing a specialized healthcare intervention or operation in a relaxed atmosphere in an environment not resembling a hospital,
- a way of accessing increasingly diverse complementary therapies related to preventive healthcare measures.

**Figure 1:**
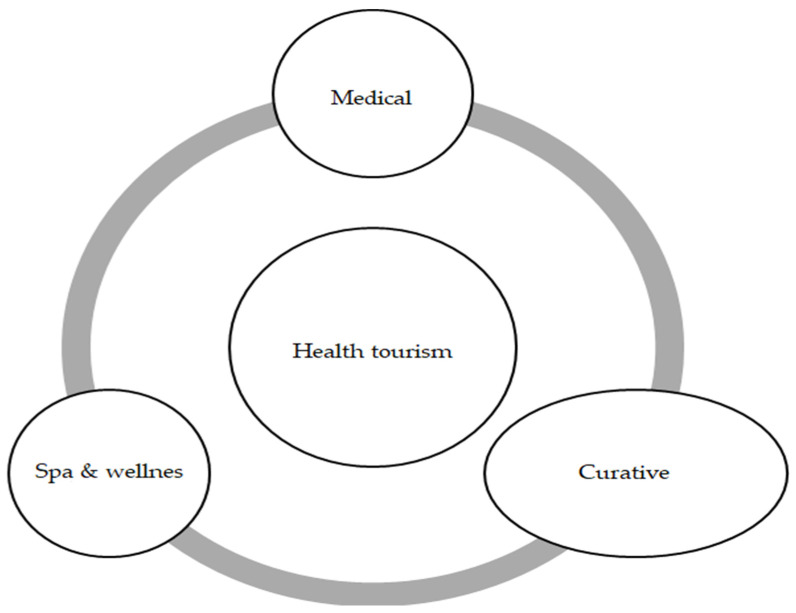
Division of health tourism. Source: [37,41].

Health tourism can be divided into the following types, as shown in Figure 1.

Health tourism has become more popular in the post-2000 era [42] because society increasingly often needs physical and mental leisure. Furthermore, there is noticeable development in the market of services for disabled tourists.

## 3. Materials and Methods

### 3.1. Research Methodology

The aim of the article was achieved on the basis of a review of the current literature on medical tourism based on the SotA procedure, as detailed by Barry et al. [43]. The study used bibliometric methods in order to facilitate the analysis of a large number of publications [44]. The authors relied on descriptive bibliometrics in analyzing the scientific research trends and in identifying the right scientists and research centers [45,46].

The following tools and techniques were used in the bibliometric analysis: analysis of changes in the number of publications; citation analysis; and the co-word method. The analysis of changes in the number of publications and citations allowed identification of the trends and determination of the levels of knowledge transfer and dissemination by representatives of different scientific centers. In turn, the co-word analysis enabled the identification of main thematic areas addressed in health tourism publications with the use of a cluster analysis method developed by Zhu et al. [47]. The clustering was estimated with VOSviewer (Visualising Scientific Landscapes) version 1.6.18 (2022) [48], a tool for building and visualizing bibliometric networks that is capable of handling large text files with descriptions of bibliographic records from well-known databases, including the Web of Science (WoS).

### 3.2. Data Collection and Research Tasks

The data used in this analysis were retrieved from the Web of Science database on November 15, 2022. Web of Science is one of the major search engines for scientific sources, and it offers a wide variety of documents. The fundamental issue in searching for records is to identify the keywords believed to be of relevance for the problem concerned [49]. The analysis covered papers with the following expressions in their titles: “health tourism,” “medical tourism,” “spa tourism,” and “spa and wellness.” Only articles that were grouped in the "title" section have been addressed.

This resulted in retrieving a set of 1533 publications, which were then subject to a refined selection process based on the following limitative criteria:

(1) date published: the study took account of papers published between 2000 and 2022;
(2) publication type: the study took account of papers published in reviewed scientific journals and books;
(3) publication subject: the study took account of publications focused on selected keywords.

After applying the limitative criteria, the dataset comprised 1493 publications.

As the next step, the authors tried to discover the general trend in the number of publications and citations, and to identify the main researchers, centers, countries, journals, and research areas related to health tourism.

## 4. Results

### 4.1. General Trend in Health Tourism Publications

**Figure 2:**
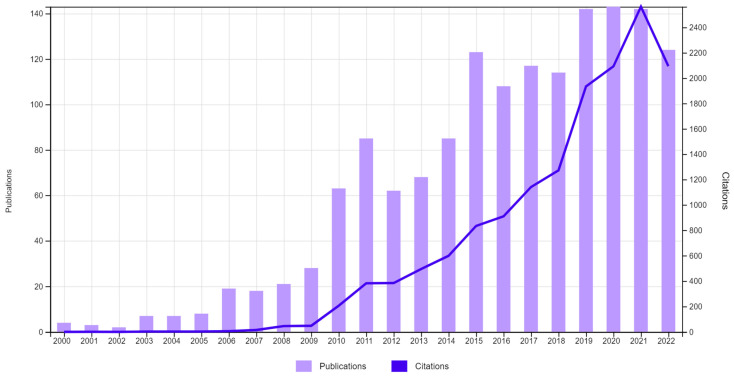
Number of health tourism publications from 2000–2022. Source: own elaboration based on the dedicated database.

Figure 2 presents the number of WoS publications addressing health tourism from 2000–2022. Note that the interest in the topic covered by this analysis follows a steady growth trend. Three sub-periods of development of health tourism publications can be identified: (1) small interest: 2000–2009; (2) medium interest: 2010–2018; (3) high interest: 2019–2022 (with an average of 140 papers per year). The significant growth in the number of publications over the last years can be explained by greater care in addressing health tourism issues.

The growing interest in health tourism topics justifies the need for a review with a structured approach to the most recent literature and for identification of future areas of research in that domain.

The largest number of papers were written in English (1343). The database also included articles written in Spanish (68), German (21), Russian (10), Chinese (7), French (6), Croatian (6), Portuguese (6), Czech (5), and Italian (4).

Most publications addressed such research topics as social sciences, business economics, and public environmental occupational health (Table 2).

Table 2 suggests that the authors represented various fields of research. However, a large number of publications addressed topics related to medical sciences, e.g., public environmental occupational health, healthcare, and internal medicine.

### 4.2. Web of Science Categories

The next step consisted of dividing the scientific publications into Web of Science categories (Table 3).

Most publications (as many as 411) fell into the category of “Hospitality Leisure Sport Tourism”.

### 4.3. Analysis of Publication Sources

The most popular journals with health tourism papers include *Sustainability, Tourism Management, International Journal of Environmental Research and Public Health, Iranian Journal of Public Health*, and *International Journal of Healthcare Management* (Table 4).

The most popular publishers include Taylor & Francis, Elsevier, Springer Nature, and Emerald Group Publishing (Table 5).

The next step was to present publication authors, their countries of origin, and their affiliations.

### 4.4. Analysis of Publications by Country and Research Center

The greatest number of health tourism publications were authored by Jeremy Snyder (Table 6).

Most authors of health tourism publications originate from the United States and China. Many other publications were related to authors coming from countries such as Malaysia, United Kingdom, and Canada (Table 7).

The next step focused on analyzing the research centers. Note that research on health tourism was highly dispersed, with the largest number of papers published by employees of Simon Fraser University (Table 8).

The authors also represented the State University System of Florida, the Ministry of Education Science of Ukraine, and the University of London.

### 4.5. Analysis of Main Research Areas

The next step in identifying the research areas related to health tourism was the co-word analysis, which served as a basis for the subsequent cluster analysis. Note that the co-word or co-occurrence analysis is a technique that allows examining the actual content of a publication [50]. It uses words derived from the keywords defined by the author(s), and it can also be employed in analyzing words contained in the paper’s keywords, title, abstract, or index, and even in its full text [51]. As a consequence, a thematic relation can be established between frequently co-occurring words, which allows identifying thematic clusters and outlining the trends of future research areas.

The co-word analysis was performed as follows:

1. Retrieving database records using criteria detailed in the Methodology section.
2. Exporting data, including authors’ names, title, abstract, keywords, and references.
3. Mapping the relationships that underpin the thematic clusters. The analysis of frequencies was carried out for a set of keywords that occurred in no less than ten phrases.
4. Analyzing the results.

**Figure 3:**
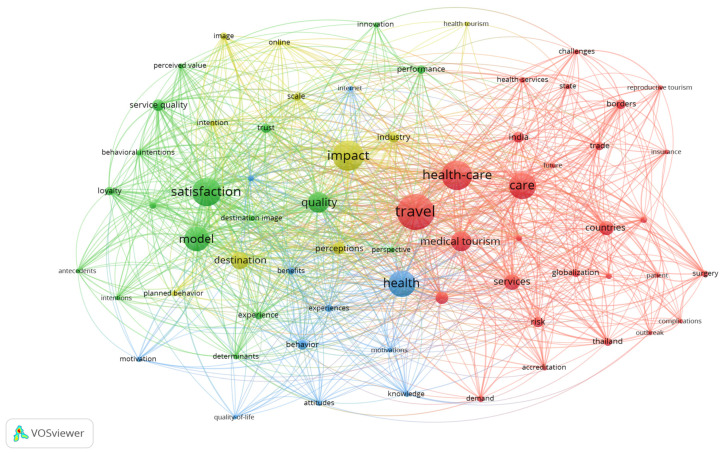
Co-Word Cluster Map. Source: own elaboration based on the dedicated database.

Figure 3 presents the visualization of keywords for the “health tourism” thematic area, with “travel”, “health-care”, “care”, “health”, “satisfaction”, “impact”, “model”, “quality” and “destination” as the most frequent occurrences.

The co-word analysis identified four research clusters related to the topic of health tourism (Figure 3).

Cluster 1 (green): patient satisfaction built upon trust; Cluster 2 (yellow): health impacts of the destination; Cluster 3 (blue): health behavior as a major part of human activity; and Cluster 4 (red): traveling with a view to regain one’s health.

## 5. Discussion

### 5.1. Cluster 1 (Green): Patient Satisfaction Built upon Trust

The first cluster covered by the analysis dealt with medical sciences because it was related to patient satisfaction built upon trust in healthcare institutions. Highly interesting research on these matters was presented in a paper by Khodadad Hosseini and Behboudi [52]. Their goal was to examine the impacts of brand trust on a population of healthcare service users. Nowadays, healthcare managers and activists tend to increasingly rely on marketing and branding measures in order to attract and satisfy their customers. Hence, the study focused on a conceptual model designed to assess brand trust and the impact of brand image on customer satisfaction. Data were sourced from 240 survey questionnaires. The study found the following to be the most efficient aspects with the greatest impact on customer satisfaction and use of healthcare services: brand image; personnel’s sincerity in handling patients; and interactions and relationships with doctors. The authors believe that identifying important elements related to healthcare branding helps healthcare managers and operators create and protect their brands and, as a consequence, drives an increase in profitability due to greater consumer satisfaction.

Highly interesting findings on how to build patient satisfaction were brought by Liu, Ching-Yick Tse, and He [53]. The purpose of their study was to compare the impacts of health-related corporate social responsibility (CSR) factors on the intents of casual restaurant customers in the U.S. and China. They adopted an approach based on survey questionnaires to collect data in both countries. A total of 828 complete answers were used to validate the hypotheses through the modeling of structural equations. The results revealed some considerable differences in replies between the two countries. The research framework underpinning health-related CSR aspects, critical variables, and relationships among them was subject to a theoretical test and verification procedure. From a practical point of view, these findings allow the management to develop efficient (yet different) market strategies in order to promote CSR initiatives among consumers with a different cultural, political, and economic background (such as the U.S. and China) in order to increase financial benefits while building consumer satisfaction and loyalty. This is one of the few empirical studies on the impacts of consumer decision-making factors on culinary behaviors based on how the restaurants present their health-related CSR initiatives in countries with different market environments.

### 5.2. Cluster 2 (Yellow): Health Impacts of Holiday Destinations

In recent years, there has been a noticeable increase in the number of people traveling for health reasons [54,55,56,57,58]. Many scientists indicate that domestic and international health destinations are attractive mostly because of the differences in prices of products and services offered [59,60,61,62]. According to [62,63], a broad range of tourism services and products—combined with the patients and their accompanying persons having an enjoyable stay in a tourist destination—has a clear positive effect on their health. Another aspect of importance to tourists [63] is the destination itself and its surroundings.

One of the very few positive consequences of the COVID-19 pandemic is people becoming environmentally committed and interested in nature. An interesting study on this was presented by Allison Williams and Rannveig Ólafsdóttir (2022), who indicate that traveling has become possible again, and, thus, people can restart using healthcare tourism services based on natural assets viewed from a therapeutic perspective. Their research suggests that the COVID-19 pandemic contributed to drawing a number of conclusions. First, people realized that they can work remotely from any location (obviously depending on the nature of their work). Second, as they rely on innovative solutions to communicate with others (and for other purposes), they started to appreciate the benefits derived from modern technologies. Third, they started to value and pay particular attention to their health. The last aspect indicated by the researchers were natural values. The COVID-19 era saw a breakthrough in tourism because the traveling restrictions and social isolation made people appreciate nature and enjoy leisure in a natural environment. Similar conclusions were presented in a case study for Poland by Wojcieszak-Zbierska et al. [64], who demonstrated that at the time of the COVID-19 pandemic, many people decided to spend their free time on agritourism farms because they found it to be the right option for them and, most of all, to be a safe form of leisure. The study also highlighted that staying on agritourism farms had a beneficial impact on the visitors’ recovery and health. Another important aspect addressed by Xiang Yan and Shenjing He [65] is the way of financing a stay, which is expected to improve one’s physical and mental health. The authors noted that tourists increasingly often opt for staying in an attractive location, which is supposed to meet their specific individual needs related to maintaining a good physical and mental state while having a thrilling experience. The thing that matters to tourists is the destination, whereas financial resources are often a secondary concern.

As shown by the analysis of international papers, including by Aikaterini Manthiou, Volker G. Kuppelwieser and Phil Klaus; Agapito, D., Mendes, J. and Valle, P.; Cetin, G., Bilgihan, A. [66,67,68], respectively, the location of a tourist destination is of tremendous importance to the visitors’ health. This is mostly due to the growing value and importance of needs (especially including higher-order needs) and changes in the structure of the population’s needs and preferences that have been witnessed over the last ten to twenty years. These developments largely affect today’s social consumption model, which triggers changes in the service market and in the production and supply of goods. There is continuous growth in the capacity to meet a broad range of needs, and consumers keep changing their inclinations, preferences, and even habits. In turn, it follows from a study by Hung, W. L., Lee, Y. J., Huang, P. H.; Rodríguez Molina, M. Á., Frías-Jamilena, D. M., Castañeda-García, J. A. [69,70], respectively, that consumer expectations are no longer limited to having a place to rest. Visitors also want their destination to add value through positive emotions, experiences, education, and improved health.

### 5.3. Cluster 3 (Blue): Health Behaviors as an Important Part of Human Activity (Including the Economic Aspect, Which Plays a Decisive Role in Choosing a Tourism Destination)

Today’s lifestyle concept attracts constant interest from researchers around the world [71,72,73,74,75]. Its definition encompasses the whole range of an individual’s characteristic daily behaviors, which express his/her personality traits. The concept has strongly gained in popularity over recent years because of intense changes in two areas of human life: health and consumption of goods and services. As rightly noted in a number of studies, including by Han H. and Heung V., Kucukusta D., Song H. [76,77], respectively, currently, people can be observed to attach greater importance to their own safety and wellbeing when traveling and upon arrival at a holiday destination. This can be explained largely by the COVID-19 pandemic situation, which has certainly reinforced the changes in health habits related to the adherence to hygiene and sanitary standards at tourism destinations. Health behaviors mean those related to human health; the literature on the subject divides them into health-promoting and self-destructive actions. A number of researchers, including Hofer S., Honegger F., Hubeli J. and Hopkins L., Labonte R., Runnels V., Packer C. [78,79], respectively, emphasize that in order to discuss the changes in health-related behavior, it is necessary to gain in-depth knowledge of the underlying mechanisms. As there is growth in demand for diverse forms of health tourism (including medical tourism), there is also a restructuring of the tourism product offered. According to Białk-Wolf, A., Arent, M., Buziewicz, A. and Alejziak W. [27,80], respectively, many tourists today realize the positive role of physical activity. For a modern human, proper nutrition habits, a positive mental attitude (especially after the aggravation of the COVID-19 pandemic), reliable information, preventive healthcare, and physical activity itself play an important role in improving his/her living conditions. Hence, health behaviors are undoubtedly a major part of today’s human activity.

In turn, another issue was addressed by Forgione DA, Smith PC.; Bagozzi, R. P., Gurhan-Canli, Z. and Priester, J. R.; Lam, T. and Hsu, C. H. C.; March, R. and Woodside, G. [81,82,83,84], respectively. who found that demand- and supply-side changes in today’s tourism market are driven by changes to the tourist’s purchasing behavior. According to them, there is change in the forms of travel organization; in the quality, duration, and frequency of traveling; and in the ways and forms of spending free time. Consumers increasingly often opt for leisure scenarios that involve physical activity. Many researchers also focused their attention on one more aspect. Namely, according to Hudson S., Xiang R.L.; Heather Hartwell, Alan Fyall, Cheryl Willis, Stephen Page, Adele Ladkin, Ann Hemingway; Allison Drinkert, Neha Singh; Ediansyah, Mts Arief, Mohammad Hamsal, Sri Bramantoro Abdinagoro; Yingru Li, Lin Liu, Jianguo Chen, Jiewen Zhang [85,86,87,88,89], respectively, despite society becoming increasingly aware of the importance of healthcare, there still is need for social education on how to take care of one’s health condition. In turn, Chihiro Morito and Sunildro LS Akoijam, Tabassum Khan [90,91], respectively, note that building adequate levels of awareness of one’s own health behaviors is of key importance, as it drives health-promoting attitudes. Health behavior is also related to a social and cultural context [91]. Culture has an impact on a number of aspects, including the standards of living and lifestyle of a community, how much they know about their health habits, and how they perceive their health [92,93,94]. The use of media in shaping health-promoting attitudes also plays an important role. The technological and technical development, combined with state-of-the-art social messengers, is what makes media an important stream of information, including about health. Hence, social campaigns ran on the TV, radio, FB, and Instagram are designed to make the information reach a wide audience [95,96,97,98,99].

### 5.4. Cluster 4 (Red): Traveling with a View to Regain One’s Health

Tourism is a form of physical activity [100,101,102,103,104,105] that consists in traveling away from one’s place of permanent residence to rest or explore. It includes business trips, as well as holiday, health, and other travels with accommodation away from home. According to the literature on the subject, health tourism means curative tourism, spa and wellness tourism, and medical tourism [106,107]. As shown in a study by Kachniewska [108], it develops in response to today’s social and demographic changes (including needs related to diseases of affluence and ageing societies), while also triggering the potential for new consumer needs and trends. An interesting aspect was also presented in studies by Saint-Pierre, C.; Herskovic, V.; Roberts, F.S.; Darbellay, F.; Stock, M.; Neil Lunt, Percivil Carrera; Tze-Jen Pan, Wen-Chang Chen [109,110,111,112,113,114], respectively, who indicated a new trend emerging in health tourism. It suggests that in addition to products and services, tourists also buy the accompanying experiences, emotions, and sensations when traveling. According to the researchers, the consumers’ health-oriented trips should be connected to a holiday destination that offers appropriate values (e.g., environmental benefits: clean air, favorable climate, mineral waters, etc.), while also delivering some components that affect emotions. Traveling is supposed to be interesting, pleasant, and engaging. Therefore, studies by Pearce, P.L; Buda, D. [115,116], respectively, reveal yet another crucial factor that guides health-oriented trips. The authors mostly focused on explaining the roles and importance of sophisticated technologies and techniques, which make it possible for today’s consumers to travel long distances with the use of state-of-the-art tools.

Nowadays, people expect to be able to relax in a healthy and active way in urban and rural areas. The tourism sector is currently focused on offering a customized portfolio composed of medical, spa, wellness, and other services [117,118,119]. People travel to clinics, spa resorts, and sanatoriums (go on therapeutic trips) for different reasons and want to improve their health status by undergoing professional rehabilitation or treatment programs [120]. An important topic addressed by Neil Lunt and Percivil Carrera was the context of how medical tourists finance their travels. According to them, some tourists rely on social health programs, while others use their own resources (pay out of pocket for accessing dentist, beauty, and programmed treatments). The authors indicate that as a consequence of administrative and legal regulations, tourists very often rely on their own funds in paying for medical services (especially in Europe), which can be viewed as a financial disharmony. They also note that there is still confusion as to the rights of patients who travel abroad, e.g., in order to receive a treatment.

## 6. Conclusions

The recent years have seen growing interest in and importance of health tourism. A healthy lifestyle, as currently developed around the globe, is at the very core of today’s social changes. It promotes physical and mental fitness; determines the condition of an individual’s body; affects people’s pace of work, efficiency, and mental capacity; and conditions their creativity and ability to take action.

This paper was a review of the current literature on medical tourism. It provided a basis for identifying four research clusters spanning the following content: patient satisfaction built upon trust; health impacts of the destination (including the economic aspect, which plays a decisive role in choosing a tourism destination); health behavior as a major part of human activity; and traveling with a view to regain one’s health. This study provides grounds for some conclusions. First, health tourism contributes to improvements in individuals’ mental and physical health; in that context, an important role is played by the behavioral and emotional dimension and by the experience lived in a tourism destination. Another important aspect to humans is the destination itself and its surroundings, where visitors can undergo a variety of medical and curative procedures and—first of all—take care of their health and recover their vitality. Health-oriented trips, especially in the era of the COVID-19 pandemic, changed the way tourists behave when their own safety is concerned. It means that they expect their hosts to guarantee a safe and peaceful experience during their stay.

The authors realize certain restrictions affecting this study, but believe that a further analysis would provide valuable grounds for continued in-depth scientific research. Indeed, there is a great need for more research on health tourism based on knowledge resources shared between tourism and related sciences.

## Figures and Tables

**Table 1:** Selected definitions of health tourism found in the relevant literature.

Author(s)	Year Published	Journal/Publisher	Definition
Wolski [30]	1970	Polish Society of Balneology, Physical Medicine and Biological and Climate Sciences	“An informed and voluntary travel away from one’s place of residence in his/her free time with a view to recover his/her body through physical and mental activities”
Goodrich [31]	1993	Journal of Travel Research	“Health tourism is defined as purposeful actions taken by tourism facilities (e.g., hotels) or destinations (e.g., Baden in Switzerland, Bath in England) to attract tourists by promoting healthcare services and facilities in addition to usual tourism amenities”
Tabacchi [32]	1997	Inter-American Travel Congress	“Any kind of travel which makes the traveler or his/her family feel healthier”
Gaworecki [33]	2003	Polish Economic Publishers	“An informed and voluntary travel away from one’s place of residence in his/her free time with a view to recover his/her body through physical and mental activities”
Bennet, King, Milner [34]	2004	Journal of Vacation Marketing	“Any kind of pleasure tourism which includes a stress-alleviating experience”
Lewandowska [35]	2007	Scientific Publishing House of the University of Szczecin	“Services which address health and leisure-related needs, improve people’s health and make them feel better”
Białk-Wolf [36]	2010	Publishing House of the Academy of Tourism and Hotel Management in Gdansk	“All relationships and developments deriving from stays and travels of people whose main motivation and goal is to improve or maintain their health status or heal their diseases”
Łoś [37]	2012	“Economic problems related to services,” Scientific Journals of the University of Szczecin	Health tourism can be divided as follows: (a) spa tourism offered in spa destinations, related to providing spa treatment services, including chronic disease management, recovery, disease prevention, and health education and promotion; (b) spa and wellness: the main goal is to offer relaxation and body care services (massage, gymnastics, cryotherapy) and ensure wellbeing (fighting stress, detoxification, oxygen therapy); (c) medical tourism offered in traditional medical centers (hospitals, clinics) in order to provide healthcare services in broad terms.

Source: [30,31,32,33,34,35,36,37].

**Table 2:** Top ten research areas related to health tourism.

Field of Research	%	Number
Social Sciences	32.7	488
Business Economics	16.3	243
Public Environmental Occupational Health	11.6	174
Health Care Sciences Services	9.7	145
Environmental Sciences Ecology	7.9	119
General Internal Medicine	6.3	95
Science Technology	3.8	57
Surgery	3.6	54
Computer Science	3.4	51
Geography	2.8	43

Source: own elaboration based on the dedicated database.

**Table 3:** Web of Science categories.

Web of Science Categories	%	Number
Hospitality Leisure Sport Tourism	27.5	411
Public Environmental Occupational Health	11.6	174
Health Policy Services	7.9	119
Management	7.6	114
Economics	7.0	105
Medicine General Internal	6.3	95
Business	6.2	93
Environmental Sciences	5.1	77
Environmental Studies	4.7	70
Health Care Sciences Services	3.7	55

Source: own elaboration based on the dedicated database.

**Table 4:** Scientific journals with the largest number of health tourism publications.

Publication Titles	%	Number
Sustainability	1.8	27
Tourism Management	1.7	26
International Journal of Environmental Research and Public Health	1.6	24
Iranian Journal of Public Health	1.4	22
International Journal of Healthcare Management	1.4	21
Journal of Travel & Tourism Marketing	1.1	17
Tourism Review	1.0	15
Asia Pacific Journal of Tourism Research	0.8	13
Current Issues in Tourism	0.8	12
Journal of Travel Medicine	0.7	11

Source: own elaboration based on the dedicated database.

**Table 5:** Publishers with the largest number of health tourism publications.

Publishers	%	Number
Taylor & Francis	11.8	176
Elsevier	9.6	144
Springer Nature	9.4	140
Emerald Group Publishing	5.3	79
Wiley	4.9	74
MDPI	3.8	57
Sage	2.5	37
Oxford Univ Press	2.2	33
Lippincott Williams & Wilkins	1.8	28
Iranian Scientific Society Medical Entomology	1.5	22

Source: own elaboration based on the dedicated database.

**Table 6:** Authors of health tourism publications.

Author	Number of Health Tourism Publications	Rank
Snyder, J.	43	1
Crooks, V.A.	42	2
Johnston, R.	27	3
Connell, J.	18	4
Pacheco, M.A.D.	14	5
Turner, L.	13	6
Adams, K.	11	7
Han, H.	10	8
Ormond, M.	10	8
Rai, A.	10	8

Source: own elaboration based on the dedicated database.

**Table 7:** Number of publications by country.

Countries	%	Number
U.S.	16.7	249
China	8.8	132
Malaysia	6.0	91
United Kingdom	5.5	82
Canada	5.4	81
Australia	5.2	78
India	4.5	67
South Korea	4.3	65
Spain	4.0	60
Iran	3.5	53

Source: own elaboration based on the dedicated database.

**Table 8:** Number of publications by research center.

Affiliations	%	Number
Simon Fraser University	3.3	49
State University System of Florida	1.4	21
Ministry of Education Science of Ukraine	1.3	20
University of London	1.2	19
University of Sydney	1.2	19
University of Minnesota System	1.1	17
University of Minnesota Twin Cities	1.1	17
Islamic Azad University	1.0	16
Universidad Del Norte	1.0	15
Universiti Teknologi Mara	1.0	15

Source: own elaboration based on the dedicated database.

## Data Availability

Not applicable.
